# Evaluation of Artificial Intelligence Algorithms for Diabetic Retinopathy Detection: Protocol for a Systematic Review and Meta-Analysis

**DOI:** 10.2196/57292

**Published:** 2024-05-27

**Authors:** Jaime Angeles Sesgundo III, David Collin Maeng, Jumelle Aubrey Tukay, Maria Patricia Ascano, Justine Suba-Cohen, Virginia Sampang

**Affiliations:** 1 Office of Medical Research University of Nevada, Reno School of Medicine Reno, NV United States; 2 Kirk Kerkorian School of Medicine at UNLV Las Vegas, NV United States; 3 College of Osteopathic Medicine Touro University Nevada Henderson, NV United States; 4 Graduate Medical Education Consortium Valley Health System Las Vegas, NV United States; 5 Department of Family & Community Medicine Penn State College of Medicine Hershey, PA United States

**Keywords:** artificial intelligence, diabetic retinopathy, deep learning, ophthalmology, accuracy, imaging, AI, DR, complication, retinopathy, Optha, AI algorithms, detection, management, ophthalmologists, early detection, screening, meta-analysis, diabetes mellitus, DM, diabetes, systematic review

## Abstract

**Background:**

Diabetic retinopathy (DR) is one of the most common complications of diabetes mellitus. The global burden is immense with a worldwide prevalence of 8.5%. Recent advancements in artificial intelligence (AI) have demonstrated the potential to transform the landscape of ophthalmology with earlier detection and management of DR.

**Objective:**

This study seeks to provide an update and evaluate the accuracy and current diagnostic ability of AI in detecting DR versus ophthalmologists. Additionally, this review will highlight the potential of AI integration to enhance DR screening, management, and disease progression.

**Methods:**

A systematic review of the current landscape of AI’s role in DR will be undertaken, guided by the PRISMA (Preferred Reporting Items for Systematic Reviews and Meta-Analysis) model. Relevant peer-reviewed papers published in English will be identified by searching 4 international databases: PubMed, Embase, CINAHL, and the Cochrane Central Register of Controlled Trials. Eligible studies will include randomized controlled trials, observational studies, and cohort studies published on or after 2022 that evaluate AI’s performance in retinal imaging detection of DR in diverse adult populations. Studies that focus on specific comorbid conditions, nonimage-based applications of AI, or those lacking a direct comparison group or clear methodology will be excluded. Selected papers will be independently assessed for bias by 2 review authors (JS and DM) using the Quality Assessment of Diagnostic Accuracy Studies tool for systematic reviews. Upon systematic review completion, if it is determined that there are sufficient data, a meta-analysis will be performed. Data synthesis will use a quantitative model. Statistical software such as RevMan and STATA will be used to produce a random-effects meta-regression model to pool data from selected studies.

**Results:**

Using selected search queries across multiple databases, we accumulated 3494 studies regarding our topic of interest, of which 1588 were duplicates, leaving 1906 unique research papers to review and analyze.

**Conclusions:**

This systematic review and meta-analysis protocol outlines a comprehensive evaluation of AI for DR detection. This active study is anticipated to assess the current accuracy of AI methods in detecting DR.

**International Registered Report Identifier (IRRID):**

DERR1-10.2196/57292

## Introduction

Diabetic retinopathy (DR) has a profound presence in medicine and ophthalmology. It is one of the most common complications of diabetes mellitus (DM), attacking many areas of the body and causing issues like end-stage renal disease and cardiovascular illness [1]. DR’s pathophysiology involves hyperglycemia, loss of capillary structural integrity, inflammation, and neurodegeneration as contributors to the condition’s development; eventually, patients will notice signs of visual impairment and potential vision loss [1]. These symptoms burden many people globally, with DR having a prevalence of 8.5% among the world’s adult population [2]. Patients with diabetes have an increased DR prevalence of 34.6% [3]. The commonality of this pathology makes careful observation of DM progression and potential DR a priority [3].

DR has a wide distribution in populations, with symptoms ranging from mild to proliferative, and its impact can be detrimental to people’s lives. Thus, it is good practice for patients with diabetes to be frequently screened for clinical signs of DR. As of their 2017 update, the American Diabetes Association states patients with type 1 DM are to be initially screened for DR within 5 years of onset, and patients with type 2 DM are to be initially screened at the time of diagnosis; then, they are to be checked annually unless no clinical signs exist, in which they can alternatively be screened every 2 years [4]. As a result, physicians should be well-equipped to screen for DR in their clinics. Done traditionally with a dilated funduscopic examination, there have been increasingly more talks to include artificial intelligence (AI) in DR screening and general clinical decision-making.

AI involves the use of computers to perform tasks that typically require human intelligence. It has taken the world by storm, with industries increasingly exploring its capabilities [5]. The 2017 PwC report on AI projected that AI could significantly increase global gross domestic product, estimating an impact of US $2 trillion to US $4 trillion by 2022, and potentially reaching US $4 trillion to US $8 trillion by 2024 [6]. This growing trend sparked discussions about its potential within health care and medical subspecialties. Ophthalmology is ever growing in technology and innovation; therefore, it is of no surprise to see an initiative for AI integration into this field. Previous advancements in ophthalmic care include integrating telemedicine into DR screenings to decrease appointment wait time adherence difficulties [7]. Wilson et al [8] tackled the increasing demand for screenings by proposing teleophthalmology within primary care clinics. Now, the focus is on AI. Gulshan et al [6,9] used deep convolutional neural networks to grade 128,175 fundus images with remarkable specificity and sensitivity. Furthermore, population demographics are changing, with the median age rising globally and conditions like retinopathy of prematurity becoming more prevalent [10]. The AI revolution is finding ways to improve disease assessment and diagnosis in the field.

**Figure 1:**
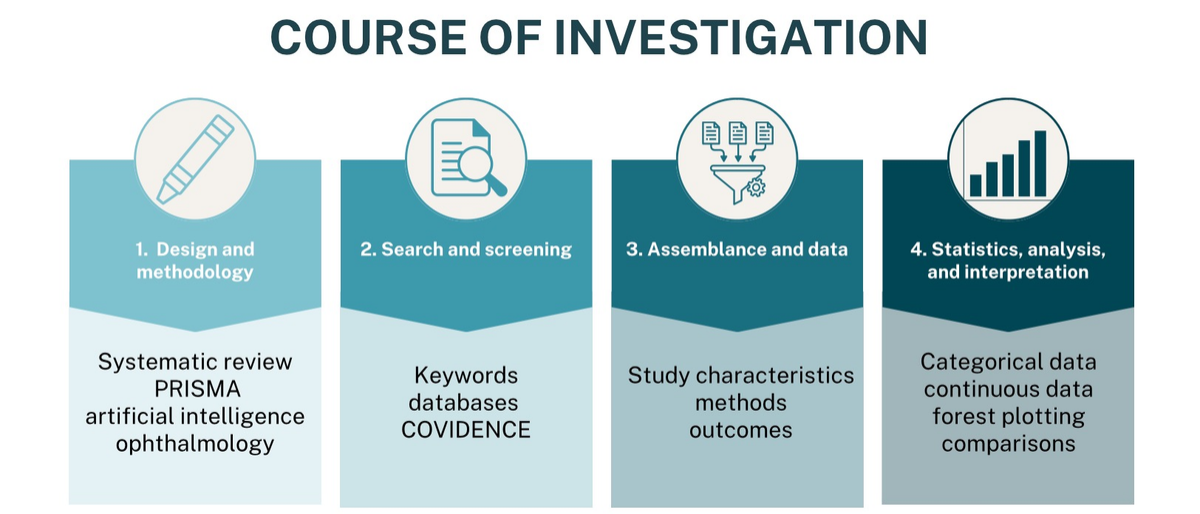
Steps for inception and conduction of the project. PRISMA: Preferred Reporting Items for Systematic Reviews and Meta-Analysis.

Following the course of action shown in Figure 1, this systematic review aims to identify the current and trending use of AI in ophthalmology and their effectiveness in diagnosing, specifically for patients with DR. Factors considered are the sensitivities and specificities of AI versus gold standard diagnostics, accuracy and reliability of mentioned modalities, and their efficiency and economics. Understanding the status of its discussion and integration will help trend industry standards and identify qualms and achievements in the current time. Overall, this systematic review and meta-analysis will critically examine the current body of knowledge regarding the use of AI methods for detecting DR and define its accuracy in comparison to ophthalmologists, or other health care professionals. In addition, this review will evaluate the potential use of AI within the clinical setting to improve DR screening, monitoring, and patient outcomes. Ultimately, this study will provide a detailed understanding of AI’s current role in ophthalmology and highlight its potential future applications.

## Methods

### Overview

A systematic review and meta-analysis will be conducted in concordance with the PRISMA (Preferred Reporting Items for Systematic Reviews and Meta-Analysis) guidelines [11]. The scope of this systematic review will examine the current body of literature and provide an update regarding the performance and accuracy of AI or machine learning algorithms at detecting DR. Published full-text papers including randomized controlled trials, controlled experiments, or cohort studies are included. Publications that are not original research in nature, such as abstracts, editorials, or review papers will be excluded. In the event of future amendments to this protocol, these changes and the date of implementation will be documented.

### Study Design

This systematic review will use multiple web-based databases and perform a quantitative meta-analysis all while following the PRISMA guidelines [11].

### Eligibility Criteria

The inclusion and exclusion criteria for the systematic review are given in Table 1.

**Table 1:** Inclusion and exclusion criteria.

Inclusion		Exclusion		
**Population**				
	Individuals 18 years or older	Individuals younger than 18 years		
	Diverse populations	Populations with specific comorbidities		
**Intervention**				
	AI^a^ methods in retinal image analysis	Studies with nonimage-based applications		
**Comparison**				
	Studies with clear reference for DR^b^ diagnosis	Studies without a direct comparison group		
	Comparisons with ophthalmologist or optometrist	N/A^c^		
**Outcome**				
	Measurement of diagnostic accuracy	Outcomes unrelated to DR detection		
**Study characteristics**				
	Peer-reviewed studies published on or after 2022	Studies published before 2022		
	Randomized controlled trials, case-control, or cohort studies	Incomplete or unclear research methodologies		

^a^AI: artificial intelligence.

^b^DR: diabetic retinopathy.

^c^N/A: not applicable.

### Search Strategy

Relevant peer-reviewed papers published in English will be identified by searching the international databases: PubMed, Embase, CINAHL, and the Cochrane Central Register of Controlled Trials. The following search queries will be used: (“Diabetic Retinopathy” AND “Artificial Intelligence”), (“Deep Learning” AND “Diabetic Retinopathy”), (“Computer Vision” AND “Diabetic Retinopathy Detection”), (“AI” AND “Accuracy” AND “Diabetic Retinopathy”), (“AI” AND “Sensitivity” OR “Specificity” AND “Diabetic Retinopathy”), (“AI Algorithms” AND “Ophthalmology Workflow”). Only papers published in or after 2022 will be included. Subsequently, the identified studies will be imported and managed as citations using the reference manager EndNote 21 (Clarivate Analytics) [12]. Furthermore, to facilitate the paper screening process, authors will use the systematic review manager software Covidence (Veritas Health Innovation) [5].

### Data Management

#### Study Selection

In the initial screening phase, 2 authors (JS and DM) will independently review the titles and abstracts of the selected studies. They will rigorously evaluate these studies against the predefined inclusion and exclusion criteria, using a designated assessment form. In cases where the selected abstracts lack sufficient information, the full texts of the studies will be retrieved and thoroughly assessed to determine their eligibility. Should any discrepancies arise between the 2 authors (JS and DM) during this process, a third author (VS) will be consulted for their perspective to resolve any disagreements. In situations where data are unclear or missing from the selected studies, the authors of those respective studies will be contacted for clarification.

#### Data Extraction

The data extraction process will be performed independently by 2 authors (JT and MA) using a standardized data protocol developed by the review authors. The extracted data will include the authors’ name, publication year, publishing journal, study location, study characteristics (research design and AI methods), and study outcomes (accuracy, sensitivity, and specificity). The standardized data collection form is summarized in Textbox 1. Any discrepancies arising during data extraction will be resolved through a discussion involving the third author (JSC).

**Components of data extraction.**

- Study characteristics
  Author, year
  Country
  Study design
  Sample characteristics
- Artificial intelligence methods
  Model
  Image modality
  Data source
- Comparator (ophthalmologist, optometrist, and manual grading)
- Outcome measures
- Quality assessment
- Notes

#### Quality Assessment

A total of 2 review authors (JS and DM) will independently perform a critical appraisal and determine the risk of bias in studies. Using the Quality Assessment of Diagnostic Accuracy Studies tool for systematic review, the methodological quality of eligible studies will be graded for risk of bias as high, low, or unclear risk [13]. Similarly, concerns regarding the applicability of studies will be graded as high, low, or unclear concern of applicability. In case of discrepancies in study evaluation, the resolution will be facilitated by a third author (VS) to reach a consensus.

### Data Synthesis and Statistical Analysis

Upon completion of the systematic review, data synthesis will be considered. If among the selected studies, there is sufficient homogeneity for comparison, a meta-analysis will be performed. This will consist of a quantitative approach. The quantitative synthesis will comprise a random effects meta-analysis model to account for between-study variability in addition to within-study variability. Data summarization for dichotomous outcomes will be displayed using odds ratio, relative risk, or hazard ratio with their respective 95% CIs. Data summarization for continuous outcomes will be displayed using the standardized mean difference with 95% CI.

Heterogeneity will be assessed quantitatively using the *I*^2^ statistic and chi-square test. Values of *I*^2^ greater than 50% will indicate substantial heterogeneity and will be further explored. To address publication bias, funnel plots will be created for each meta-analysis containing 10 or more studies. Any asymmetry in plots will be investigated with the Egger test. If publication bias is detected, the trim-and-fill method will be used to estimate the effect of bias on meta-analysis results.

The statistical analysis will be performed using the Review Manager (RevMan) software (version 5.9.7; The Cochrane Collaboration). For statistical processes not supported on RevMan, STATA (StataCorp) will be used.

### Ethical Considerations

The study will involve a secondary analysis of research data. All data compiled from the web-based databases and used for the study are open-access and available to the public. As such, institutional review board or ethics committee approval was not required. Study data will be deidentified to ensure no use of individually identifiable health information.

## Results

With the results of this systematic review and meta-analysis, we provide key insight into the trends and advancements in AI algorithms for DR detection, assessing their diagnostic accuracy, sensitivity, and specificity compared to traditional ophthalmological evaluations. We expect to highlight the potential of AI to enhance DR screening and management, emphasizing improvements in efficiency and cost-effectiveness.

Using selected search queries across multiple databases, we accumulated 3494 studies on our topic of interest, of which 1588 were duplicates, leaving 1906 unique research papers to review and analyze. The methods and procedures are conducted in concordance with the PRISMA guidelines, quality assurance is guaranteed with 2 review authors (JS and DM) independently appraising each study, and the process is expected to be completed by the end of January 2025.

## Discussion

### Principal Findings

DR is one of the most common complications of DM and affects a significant portion of the population. Patients with diabetes should be screened annually for DR. In general, diabetic retinopathy is diagnosed using a dilated fundoscopic examination, and there are discussions about integrating AI, an exponentially growing innovation, into the practice. With an ever-aging population, researchers and clinicians are looking for ways to improve the standard of care. As this is a protocol outlining a study that is yet to be completed, direct comparisons with existing research are not currently available. Future iterations of this work will include a comprehensive comparison with prior studies to contextualize our results within the existing body of literature on AI applications in DR screening and management. This study will look into the sensitivities and specificities of DR detection using AI and compare the results to ophthalmology-trained clinician interpretations. This study will also analyze the efficiency and economics of the tool. It will be possible to obtain all these data when performing a thorough systematic review following the PRISMA guidelines. Key Boolean pairs like “Diabetic Retinopathy” and “Artificial Intelligence” will be used to filter in publications useful for the study.

We expect to find multiple growing trends. For one, the quality of AI is expected to have improved significantly within the last 2 years. Parameters to be improved include the sensitivity and specificity of DR detection in fundoscopy along with its accuracy and reliability. This expectation would match increasing demands for AI use and regulation in our society. Furthermore, the use of AI in DR detection is expected to perform comparably to gold-standard diagnostics, if not better. In addition, the cost of using AI in clinics is expected to have decreased in recent years and be a more cost-effective alternative to the status quo. These expected findings will follow the trend of previous systematic reviews looking into AI performance in health care.

### Limitations

Although our goal with the study is to provide a current update on the status of AI in DR screening, the extent of data extraction is limited to English-written papers, leaving out data from papers written in other languages. Our study’s scope is confined to the selected search queries and databases. Therefore, the data compiled will not be truly comprehensive. The final systematic review and meta-analysis may encounter further limitations in data homogeneity and study quality across the diverse array of research papers. Furthermore, the data cannot necessarily inform clinicians how to specifically use AI in their own practice but rather provide an overarching trend of AI capabilities and usage within the field of ophthalmology.

### Strengths

AI continues to grow in effectiveness and utility; therefore, updates on its trend have been made constantly. This study will provide the most up-to-date findings on AI’s ability to screen for DR. The use of large-scale databases and multiple variations of search criteria will help filter in the most relevant publications and become very thorough in nature.

### Conclusions

This systematic review will review the most up-to-date data on AI functionality in ophthalmic DR screenings. We expect the data to portray AI as a growing and more accurate tool for ophthalmic care. Through analysis of the current state of AI in ophthalmology, a realization of AI’s current and future role in the field will be better understood. In turn, the discovery of trends would help choose decisions on how to evolve clinical practices hereafter.

## Abbreviations

AI: artificial intelligence
DM: diabetes mellitus
DR: diabetic retinopathy
PRISMA: Preferred Reporting Items for Systematic Reviews and Meta-Analysis
